# Remarks of Dr. J. McF. Gaston, Jr., at the Banquet of the Southern Medical Society

**Published:** 1893-04

**Authors:** 


### REMARKS OF DR. J. McF. GASTON, JR., AT THE BANQUET OF THE SOUTHERN MEDICAL SOCIETY.

The first step of a college graduate is usually the choice of a profession. In view of his classical and scientific studies, he generally selects one of the learned professions—teaching, law, medicine, or the ministry.

Again, the choice is restricted by circumstances between these. Many a college student goes into law and medicine because his father is a lawyer or doctor. Many a college stu-student studies medicine for the reason assigned by the great J. Marion Sims: he is no speaker and will not be a lawyer; has no patience to teach, and no spirit to preach. To these, medicine may become, as it did to Sims, a happy hit. But there are many who select medicine for other reasons, though these may also have their weight even with them. They have seen some ideal doctor. They have seen him working faithfully day and night in the interests of his profession. They have seen his cheerful smile and felt his hearty hand-shake. They have seen the results of his toil. They have heard him talk enthusiastically, not over the money he has made, but over the intracacies of a case which has just terminated successfully. They have seen him, not withholding, but publishing to the world some important discovery. They have noticed the bond of sympathy connecting patient and physician. They have been struck by the respect and confidence reposed in the family physician. They have noticed the majority of physicians living in comfort, and the few physicians who have any eye for business, living in luxury. They have also seen the peculiar drawbacks under which this ideal physician labored. They have seen his patients delay to pay his bill until they forgot they had been sick; and, then they have seen him roused at any time of night and in any weather. They have seen him uncomplainingly give up some cherished plan and the time he thought he could call his own.

They have probably read the autobiographies of such surgeons as Gross and Sims, pondered over the lives of such scientists as Pasteur, Jenner and Flint, admired the work of such living benefactors as Battey and others. They have compared the work of such men with that of the greatest literati, legislators, or ministers, and have seen the result of the comparison by no means unfavorable. They have thought of the Divine Physician going about doing good. They have prayed for his intercession in guiding aright their selection of a profession second only to that of saving souls and closely allied to it in doing that. They have thought of the field open to them for the display of journalistic, didactic, or practical talent. Irrefutable proofs have been shown them that the medical profession has lengthened the short span of man's earthly sojourn, alleviated pain, withdrawn the germs of disease, pestilence, and woe.

To the objections interposed, they answer that there is room at the top, that all the professions are crowded, that they are young and able to stand the severe tests upon their strength and patience, that their mission is to seek misery, not to avoid it ; to fight the battle of life, not to be carried through life "on flowery-beds of ease."

Such college graduates have studied medicine after such reflections. Having entered it with the determination to succeed, they have thought of nothing else. The science of medicine has unfolded the trained faculties of their minds still more ; has taken possession of their energies, and has caused them to exclaim : "The proper study of mankind is man."

The lectures of the professors are listened to as eagerly as the revelations of the text-book were perused. The demonstrations of truth are enthusiastically watched.

Such students, having secured their diplomas and won the proud title of Dr., do not end their studies, but begin them with renewed zeal. They read all the new phases of the sciences of medicine and surgery in the medical journals ; they seek to apply them to imaginary if not real cases. They do not lose the time in which they wait for practice. The practice finally comes, and though the life is indeed a hard struggle and full of bitter rebuffs, still the enthusiasm of youth conquers all. The complete preparation is found to solve many a difficult case, while the training at college is found to make every step easier. But still much remains to be done even in the little time a busy practitioner can spare for study. The field for charity is boundless, the field for faith and hope well nigh so. Fame of the most lasting kind is not beyond their reach.

